# Tubular supramolecular alternating copolymers fabricated by cyclic peptide–polymer conjugates†

**DOI:** 10.1039/d1sc02389f

**Published:** 2021-06-03

**Authors:** Qiao Song, Andrew Kerr, Jie Yang, Stephen C. L. Hall, Sébastien Perrier

**Affiliations:** Department of Chemistry, University of Warwick Coventry CV4 7AL UK s.perrier@warwick.ac.uk; Shenzhen Grubbs Institute, Southern University of Science and Technology Shenzhen 518055 China; Warwick Medical School, University of Warwick Coventry CV4 7AL UK; Faculty of Pharmacy and Pharmaceutical Sciences, Monash University Parkville VIC 3052 Australia

## Abstract

Supramolecular copolymers are an emerging class of materials, which bring together different properties and functionalities of multiple components *via* noncovalent interactions. While it is widely acknowledged that the repeating unit sequence plays an essential role on the performance of these materials, mastering and tuning the supramolecular copolymer sequence is still an open challenge. To date, only statistical supramolecular copolymers have been reported using cyclic peptide–polymer conjugates as building blocks. To enrich the diversity of tubular supramolecular copolymers, we report here a strategy of controlling their sequences by introducing an extra complementary noncovalent interaction. Hence, two conjugates bearing one electron donor and one electron acceptor, respectively, are designed. The two conjugates can individually assemble into tubular supramolecular homopolymers driven by the multiple hydrogen bonding interactions between cyclic peptides. However, the complementary charge transfer interaction between the electron donor and acceptor makes each conjugate more favorable for complexing with its counterpart, resulting in an alternating sequence of the supramolecular copolymer. Following the same principle, more functional supramolecular alternating copolymers are expected to be designed and constructed *via* other complementary noncovalent interactions (electrostatic interactions, metal coordination interactions, and host–guest interactions, *etc.*).

## Abstract 2

Tubular supramolecular alternating copolymers using cyclic peptide–polymer conjugates are synthesized by the introduction of an extra complementary noncovalent interaction.

## Graphical abstract

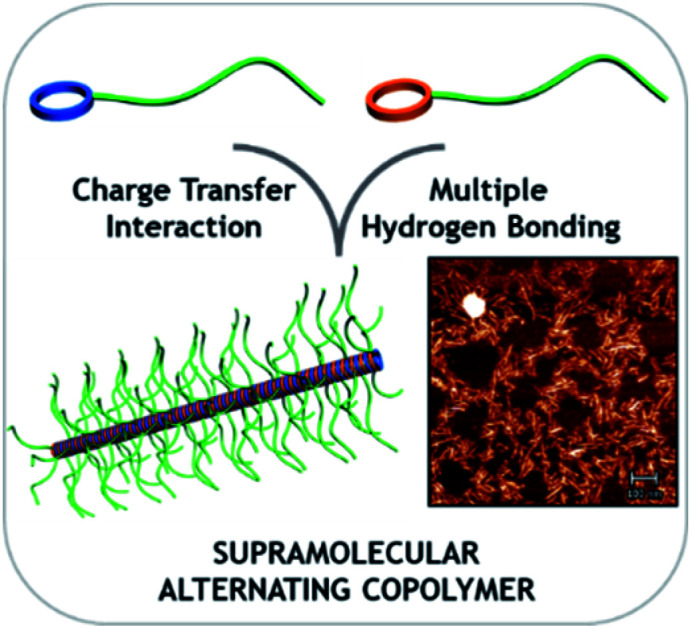

## Introduction

Supramolecular polymers are defined as polymeric arrays of monomeric units that are held together by highly directional and reversible noncovalent interactions, resulting in polymeric properties in solution and bulk.^1–9^ Nowadays, a variety of noncovalent interactions, including multiple hydrogen bonding, metal coordination, host–guest interaction, and aromatic stacking, have been utilized as driving forces to construct supramolecular polymers. The polymeric structures and reversible noncovalent interactions endow these supramolecular polymers with not only traditional polymeric behaviours, but also dynamic properties, such as stimuli-responsiveness, environmental adaptivity, and self-healing capacity.^10–14^ Moreover, the fabrication of supramolecular copolymers by simply co-assembling different monomeric units *via* noncovalent interactions allows the integration of different functionalities for various applications.^15–18^ While sequence is believed to play a crucial role on the property of supramolecular copolymers, the inherent lability of noncovalent interactions makes it challenging to construct sequence-defined supramolecular copolymers. Different strategies, including nucleation-elongation processes, kinetic control, competition, pathway complexity, and supramolecular monomer design, have been developed to fabricate supramolecular copolymers with various ordered sequences, which could be classified as block, periodic, and alternating copolymers rather than statistical copolymers.^19–22^ Among those, supramolecular alternating copolymers were the first category of supramolecular copolymers reported by Lehn and co-workers in 1990.^23^ Noncovalent interactions involving complementary units are normally used as driving forces to fabricate supramolecular alternating copolymers, such as hydrogen bonding interactions, charge transfer interactions, and electrostatic interactions.^24–31^

Supramolecular polymers assembled by cyclic peptide–polymer conjugates belong to a relatively new class of self-assembled supramolecular polymers.^32–36^ The alternating d- and l-amino acid configuration within the cyclic peptide results in a flat ring-like structure. The strong multiple hydrogen bonding interactions between these cyclic peptide rings lead to the self-assembly of cyclic peptide nanotubes.^37–40^ Conjugating polymers onto cyclic peptides prevents their lateral aggregation and improves their stability and solubility, forming tubular supramolecular polymers with core–shell structures.^41–47^ Tubular supramolecular copolymers could be easily fabricated by mixing different cyclic peptide–polymer conjugates, which has been confirmed by attaching Förster resonance energy transfer (FRET) dyes onto the conjugates.^48–50^ Supramolecular statistical copolymers are expected to form as there are no differences regarding the driving forces between the two conjugates. To fabricate supramolecular copolymers with alternating sequences, an extra complementary noncovalent interaction needs to be introduced. Charge transfer interactions drive the alternate stacking of aromatic donor and acceptor chromophores, which have been used to fabricate a series of supramolecular assemblies with different architectures, including foldamers, vesicles, gels, and supramolecular polymers.^51–58^ We believe that the inherent complementary nature of charge transfer interactions in alternating placement of the donor and acceptor units offers a potential opportunity in realizing the construction of tubular supramolecular alternating copolymers.

**Scheme 1:**
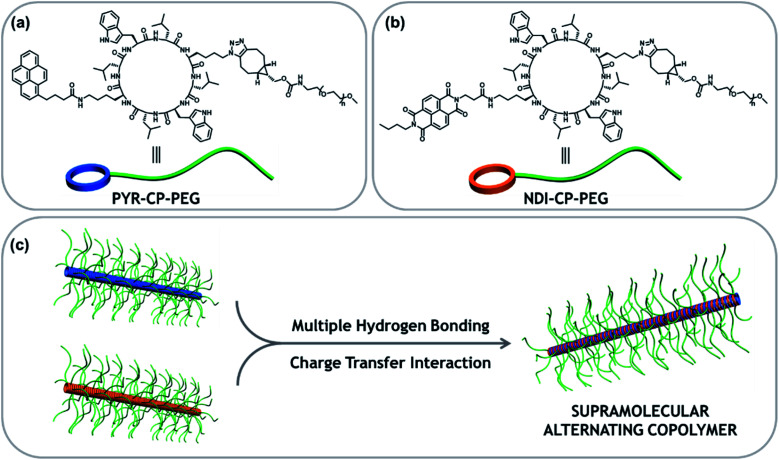
Chemical structures of PYR-CP-PEG (a) and NDI-CP-PEG (b); schematic representation showing the formation of tubular supramolecular alternating polymers based on complementary charge transfer interaction and multiple hydrogen bonding interaction (c).

Here we report the fabrication of supramolecular alternating copolymers in aqueous media driven by a combination of charge transfer interactions and multiple hydrogen bonding interactions. To this regard, two π-conjugated chromophore–cyclic peptide–polymer conjugates, one attached with an electron donor – pyrene (PYR-CP-PEG) and another one attached with an electron acceptor – naphthalenediimide (NDI-CP-PEG), are designed and synthesized (Scheme 1). PYR-CP-PEG and NDI-CP-PEG are capable of individually assembling into tubular supramolecular polymers in aqueous solutions due to the strong binding of the cyclic peptide core. When mixing PYR-CP-PEG and NDI-CP-PEG, the charge transfer interactions between PYR and NDI moieties make both conjugates more favourable of binding with their complementary counterparts, triggering the assembly of supramolecular alternating copolymers. Our findings demonstrate that the binding constant between PYR-CP-PEG and NDI-CP-PEG is as high as 7.9 × 10^5^ M^−1^, while the binding constant of PYR-CP-PEG self-association is only 3.6 × 10^4^ M^−1^. The *ca.* 20-fold difference undoubtedly supports the proposed alternating sequence. In addition, by replacing PYR-CP-PEG with a pH-responsive conjugate, the transition between supramolecular alternating copolymers and homopolymers could be reversibly controlled by pH.

## Experimental

### Materials

Fmoc-protected amino acids and coupling agents were purchased from Iris Biotech GmbH. α-Methoxy-ω-amino PEG (CH_3_O–PEG–NH_2_, *M*_n_ = 5000 g mol^−1^) was purchased from Rapp Polymere. 1,4,5,8-Naphthalenetetracarboxylic dianhydride, 1-pyrenebutyric acid *N*-hydroxysuccinimide ester, (1*R*,8*S*,9*s*)-bicyclo[6.1.0]non-4-yn-9-ylmethyl *N*-succinimidyl carbonate and other chemicals were purchased from Sigma-Aldrich. Solvents were purchased from several departmental suppliers, Honeywell, Fisher and Sigma-Aldrich.

### Characterization

#### Nuclear magnetic resonance spectroscopy (NMR)

^1^H NMR spectra were measured using a Bruker Avance III HD 400 MHz NMR spectrometer. The residual solvent peaks were used as internal references.

#### Gel permeation chromatography (GPC)

GPC was measured using an Agilent PL50 instrument with a differential refractive index detector. The instrument contained two PolarGel H columns (300 mm × 7.5 mm) and a PolarGel 5 μm guard column. DMF with 0.1% LiBr additive was used as the eluent. The system ran at 1 mL min^−1^ (50 °C), with an injection volume of 100 μL. The samples were prepared by filtering them through 0.22 μm pore size PTFE membranes, before injection. Agilent EasyVial poly(methyl methacrylate) standards were used to calibrate the instrument and output data were analysed using Agilent GPC/SEC software.

#### High-performance liquid chromatography (HPLC)

High-performance liquid chromatograms were measured using a Shimadzu Prominence HPLC, equipped with an Agilent Eclipse Plus C18 column (100 mm × 4.6 mm) with 3.5 μm micron packing. Water and acetonitrile were used as mobile phase A and B, respectively. All solvents contained 0.04 vol% TFA. The gradient used for HPLC analysis was increased from 5% to 95% B in 30 minutes. Detection was achieved *via* monitoring UV absorption at different wavelengths. Samples were dissolved in mobile phase A with concentration of 0.5 mg mL^−1^ and the injection volume was 20 μL.

#### Mass spectrometry (ESI-TOF)

ESI-TOF mass spectra were measured using an Agilent 6130B single Quad to characterize the peptides in both positive and negative ionisation modes. Samples were dissolved in methanol.

#### Ultraviolet-visible (UV-Vis) absorption spectroscopy

UV-Vis absorption spectra were measured using an Agilent Technologies Cary 60 UV-Vis spectrometer. The path length of the cuvette is 10 mm.

#### Fluorescence emission spectroscopy

Fluorescence emission spectra were measured using an Agilent Technologies Cary Eclipse Fluorescence spectrometer.

#### Small angle neutron scattering (SANS)

SANS was carried out on Larmor at the ISIS Pulsed Neutron Source (STFC Rutherford Appleton Laboratory, Didcot, UK). Prior to measurement, each sample was dissolved in deuterated solvent and placed in a 2 mm quartz cuvette. The scattering cross-section was measured over a *Q*-range of 0.004–0.5 Å^−1^ where *Q* is defined as:
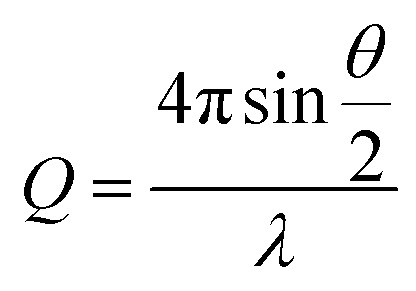
Here, *θ* is the scattered angle, and *λ* is the incident neutron wavelength.

A *Q*-range of 0.004–0.5 Å^−1^ was achieved utilizing an incident wavelength range of 0.9–13.3 Å. The detector is located 4.1 m from the sample and is 664 mm wide × 664 mm high with the beam in the centre of the detector. The beam size is 6 mm wide and 8 mm high. Each raw scattering data set was corrected for the detector efficiencies, sample transmission and background scattering and converted to scattering cross-section data (∂Σ/∂Ω *vs. Q*) using the instrument-specific software. These data were placed on an absolute scale (cm^−1^) using the scattering from a standard sample (a solid blend of hydrogenous and perdeuterated polystyrene) in accordance with established procedures.

#### Transmission electron microscopy (TEM)

10 μL of the conjugate aqueous solution was drop-casted on the carbon-coated grid. After 3 min, the solution on the grid was absorbed with filter paper. After 15 min, 10 μL of a 0.2% uranyl acetate solution was dropped onto the grid and absorbed after 30 s. Bright field TEM micrographs were obtained with a JEOL 2100Plus microscope operating at 200 kV, equipped with a Gatan OneView IS camera.

#### Atomic force microscopy (AFM)

Samples were prepared by drop-casting 2 μL of a 0.05 mg mL^−1^ solution of the conjugates onto a freshly cleaved mica substrate, which was quickly frozen and then freeze-dried. Images were collected using a Bruker-Nano Multimode V instrument in peak force tapping mode with a scan speed of 1 Hz using scanasyst-air tips (resonant frequency = 70 kHz, 0.4 N m^−1^).

## Results and discussion

### Synthesis of π-conjugated chromophore–cyclic peptide–polymer conjugates

To immobilize both the π-conjugated chromophore and hydrophilic polymer onto the cyclic peptide, chemical reactions with high efficiency and orthogonality are required. Herein, an asymmetric cyclic peptide with amino and azido groups on opposite sides (H_2_N–CP–N_3_) was designed to facilitate the orthogonal amidation reaction and alkyne/azide cycloaddition reaction. The two π-conjugated chromophore–cyclic peptide–polymer conjugates, PYR-CP-PEG and NDI-CP-PEG were synthesized by firstly attaching the corresponding π-conjugated chromophores *via* either NHS coupling chemistry or HATU coupling chemistry, followed by the attachment of the hydrophilic polymer, poly(ethylene glycol) (PEG, *M*_n_ = 5000 g mol^−1^), *via* strained alkyne/azide cycloaddition, which were thoroughly characterized by ESI-MS, HPLC and GPC (Fig. S1 and S2†). Two π-conjugated chromophore–polymer conjugates in the absence of cyclic peptide, PYR-PEG and NDI-PEG, were also synthesized as control compounds *via* amidation reaction (Fig. S3 and S4†).

### Supramolecular homopolymers formed by individual conjugates

**Fig. 1:**
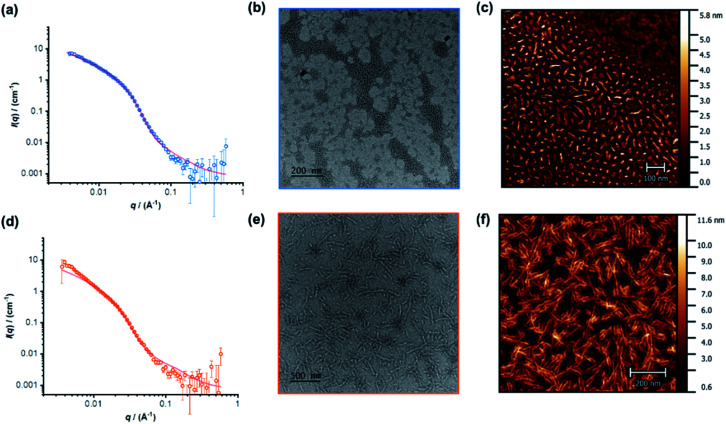
Reduced SANS scattering data and fitting to the cylindrical micelle model of PYR-CP-PEG (a) and NDI-CP-PEG (d); TEM images of PYR-CP-PEG (b) and NDI-CP-PEG (e); AFM images of PYR-CP-PEG (c) and NDI-CP-PEG (f).

The individual self-assembling behavior of conjugates PYR-CP-PEG and NDI-CP-PEG in aqueous media was investigated by small angle neutron scattering (SANS), transmission electron microscopy (TEM) and atomic force microscopy (AFM). Fig. 1a shows the reduced, corrected scattering data of PYR-CP-PEG conjugate in water. Using SASfit software, the data could be fitted using a cylindrical micelle model (Table S1†), suggesting it forming tubular supramolecular polymers in water.^59^ The formed supramolecular polymers were visualized by TEM and AFM. As presented in Fig. 1b and c, 1D structures can be evidently seen. The diameter of the tubular supramolecular polymers was narrowly distributed, while the length varied from 10 to more than 80 nm, with the average length of 36 ± 13 nm (Fig. S9a†). As for NDI-CP-PEG conjugate in water, the SANS data could also be fitted with the same cylindrical micelle model (Fig. 1d). Moreover, most likely due to the increased hydrophobicity of the NDI moiety, TEM and AFM showed the presence of much longer tubular supramolecular polymers, with the length measured to be 100 ± 31 nm (Fig. 1e, f and S9b†).

**Fig. 2:**
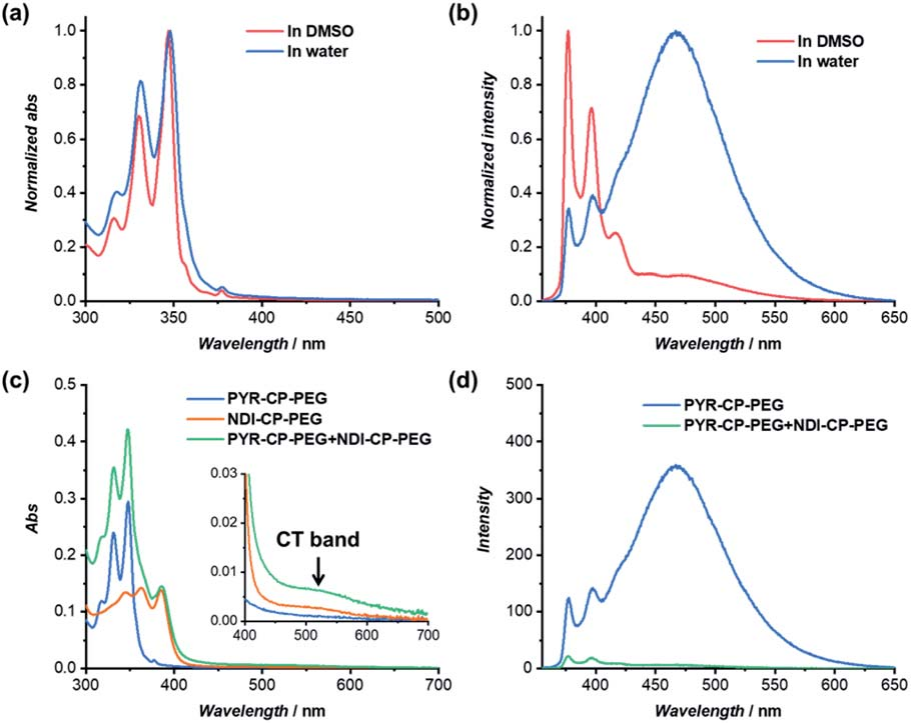
(a) Normalized UV-Vis spectra of PYR-CP-PEG in DMSO and water; (b) normalized fluorescence spectra of PYR-CP-PEG in DMSO and water; (c) UV-Vis spectra of PYR-CP-PEG, NDI-CP-PEG and PYR-CP-PEG + NDI-CP-PEG in water; (d) fluorescence spectra of PYR-CP-PEG and PYR-CP-PEG + NDI-CP-PEG in water ([PYR-CP-PEG] = 10 μM, [NDI-CP-PEG] = 10 μM, *λ*_ex_ = 335 nm).

The self-assembling behavior of conjugate PYR-CP-PEG in aqueous media was further studied by UV-Vis spectroscopy and fluorescence spectroscopy. The absorption spectra of PYR-CP-PEG were compared in both DMSO and water. As shown in Fig. 2a, a slight bathochromic shift and broadening of the absorption bands was observed when changing the solvent from DMSO to water, which is ascribed to the aggregation of PYR-CP-PEG molecules in water. A more significant difference was revealed by fluorescence spectroscopy. Characteristic emission profile of unimeric pyrene was observed in DMSO. However, in water, a new, broad emission band peaked at 460 nm appeared, corresponding to pyrene excimer. This clearly indicates the stacking of PYR-CP-PEG in aqueous environment (Fig. 2b). Meanwhile, only emission of unimeric pyrene was witnessed for the control conjugate PYR-PEG in water (Fig. S10†). All these data imply that the multiple hydrogen bonding interactions between the cyclic peptides are responsible for the formation of supramolecular polymers for PYR-CP-PEG in water, causing significant pyrene excimer emission.

### Supramolecular alternating copolymers by co-assembly of conjugates

NDI as a π-electron acceptor forms charge transfer complex (CT complex) with the π-electron donor PYR, owing to the overlap of HOMO_PYR_ and LUMO_NDI_. The CT complex between the two control compounds, PYR-PEG and NDI-PEG in water was firstly probed by UV-Vis spectroscopy and fluorescence spectroscopy (Fig. S11†). UV-Vis spectroscopy study of PYR-PEG and NDI-PEG demonstrated a new broad absorption band at 550 nm, corresponding to the charge transfer band (CT band) of the NDI/PYR CT complex. Simultaneously, emission of PYR was partially quenched, further confirming the formation of CT complex. However, the charge transfer interaction between PYR-PEG and NDI-PEG was found to be relatively weak. At a concentration of 100 μM, the fluorescence of PYR was quenched by 58.9%, while at a concentration of 10 μM, the fluorescence was only quenched by 22.8% (Fig. S12†). The binding constant between PYR-PEG and NDI-PEG could be measured by UV-Vis titration (Fig. S17†). By fitting the evolution of absorbance at 384 nm against the ratio of PYR-PEG/NDI-PEG using a 1 : 1 binding model, the binding constant was calculated to be (4.59 ± 0.05) × 10^3^ M^−1^, in accordance with the reported values.^60,61^

The complexation between PYR-CP-PEG and NDI-CP-PEG was then studied. An enhanced interaction between PYR-CP-PEG and NDI-CP-PEG is expected due to both the multiple hydrogen bonding interactions between the cyclic peptides and the charge transfer interactions between PYR and NDI. As shown in Fig. 2c, even at a low concentration of 10 μM in water, the CT band could be markedly seen by UV-Vis spectroscopy, implying a strengthened charge transfer interaction. Meanwhile, up to 96.6% fluorescence quench was observed by fluorescence spectroscopy (Fig. 2d), which might be contributed by both the strengthened charge transfer interaction and the energy transfer from PYR to the non-fluorescent CT complex within the assemblies.^62^ As a comparison, the fluorescence of PYR exhibited only 23.4% decrease when mixing PYR-CP-PEG and NDI-CP-PEG at a concentration of 10 μM in DMSO (Fig. S13†), further emphasizing the important role of multiple hydrogen bonding interactions.

**Fig. 3:**
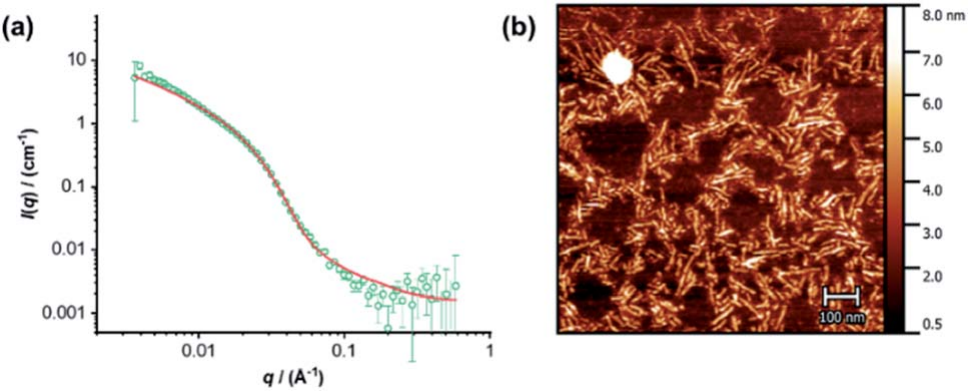
(a) Reduced SANS scattering data of the co-assembly of PYR-CP-PEG and NDI-CP-PEG. The line corresponds to a fit to the cylindrical micelle model; (b) AFM image of the co-assembly of PYR-CP-PEG and NDI-CP-PEG.

The co-assemblies of PYR-CP-PEG and NDI-CP-PEG in water were characterized by SANS, TEM and AFM. As shown in Fig. 3a, the formation of tubular supramolecular polymers in water was confirmed by fitting the SANS data of the co-assemblies of PYR-CP-PEG and NDI-CP-PEG with the cylindrical micelle model (Table S1†). 1D structures could also be seen by TEM and AFM (Fig. 3b, S14 and S15†). By analyzing the AFM images, the average length was measured to be 65 ± 20 nm (Fig. S16†). This falls in between the values of supramolecular homopolymers PYR-CP-PEG and NDI-CP-PEG, which infers their ability of forming supramolecular copolymers instead of remaining as individual homopolymers.

**Fig. 4:**
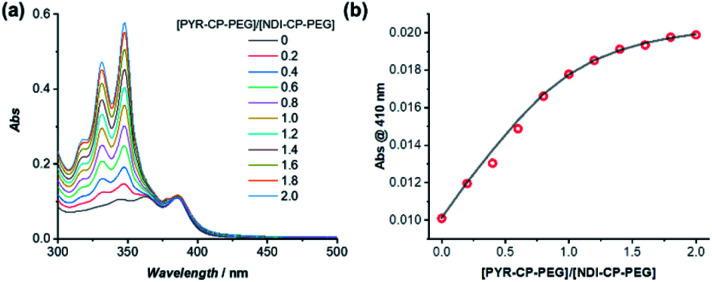
(a) UV-Vis titration of PYR-CP-PEG and NDI-CP-PEG in water; (b) evolution of absorption at 410 nm as a function of [PYR-CP-PEG]/[NDI-CP-PEG] and fitting using a 1 : 1 binding model ([NDI-CP-PEG] = 8 μM, [PYR-CP-PEG] = 0–16 μM).

PYR-CP-PEG and NDI-CP-PEG conjugates are expected to form supramolecular alternating copolymers in aqueous media, promoted by the complementary charge transfer interaction and the multiple hydrogen bonding interaction. The binding constants between PYR-CP-PEG and NDI-CP-PEG as well as the self-association of PYR-CP-PEG were measured and compared to confirm the alternating sequence in the supramolecular copolymers. Similar to the determination of the binding constant between PYR-PEG and NDI-PEG, the binding constant between PYR-CP-PEG and NDI-CP-PEG in water was obtained by UV-Vis titration and fitted using a 1 : 1 binding model. As shown in Fig. 4, the binding constant is measured to be (7.93 ± 0.88) × 10^5^ M^−1^. The more than 2 orders of magnitude increase compared to that between PYR-PEG and NDI-PEG further confirms the positive cooperativity of the charge transfer interaction between PYR and NDI and the multiple hydrogen bonding interaction between cyclic peptides. Using fluorescence spectroscopy, the binding constant of the self-association of PYR-CP-PEG in water was determined by comparing the change of pyrene unimer/excimer emission ratio at different concentrations (Fig. S18†). By fitting the data using a dimerization model, the binding constant is calculated to be (3.59 ± 1.00) × 10^4^ M^−1^, which is ∼22 times lower than that of PYR-CP-PEG and NDI-CP-PEG association. Thus, when co-assembling PYR-CP-PEG and NDI-CP-PEG in an aqueous environment, as for PYR-CP-PEG, instead of forming a supramolecular homopolymer by itself, it favors more of complexing with NDI-CP-PEG to form a copolymer with an alternating sequence. Evidence for the supramolecular alternating copolymer structure was also provided by fluorescence spectroscopy. As depicted in Fig. S19,† the copolymer formed by PYR-CP-PEG and NDI-CP-PEG showed almost no excimer emission. In contrast, a supramolecular statistical copolymer formed by PYR-CP-PEG and CP-PEG still displayed a large portion of excimer emission. The significant difference undoubtedly indicates that PYR-CP-PEG and NDI-CP-PEG co-assemble into supramolecular alternating copolymers rather than supramolecular statistical copolymers.

### Exchange dynamics

**Fig. 5:**
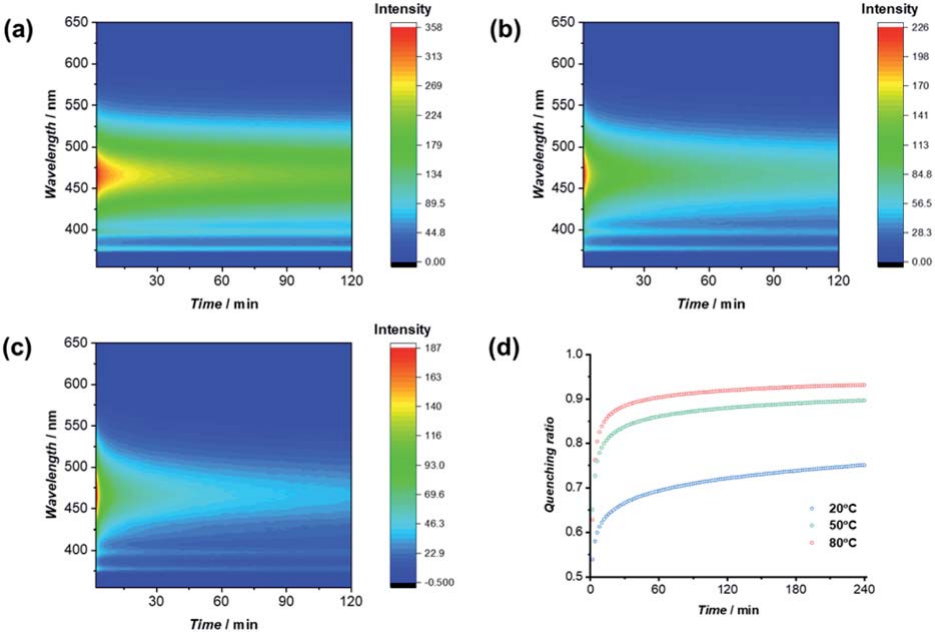
Change of fluorescence upon time at 20 °C (a), 50 °C (b), and 80 °C (c); evolution of quenching ratio *I*/*I*_0_ over mixing time at 20 °C, 50 °C, and 80 °C (d).

Utilizing the fluorescence quenching phenomenon caused by CT complexation, we could further probe the exchange dynamics of the two π-chromophore conjugates (PYR-CP-PEG and NDI-CP-PEG). In other words, by monitoring the change of fluorescence over time, we could describe the process of two individual supramolecular homopolymers co-assembling into alternating copolymers. Emission spectra, upon mixing the aqueous solutions of conjugates PYR-CP-PEG and NDI-CP-PEG in a 1 : 1 volume ratio, were taken at different time intervals under different temperatures. As clearly indicated in Fig. 5, the emission intensity decreased as a function of time after mixing PYR-CP-PEG and NDI-CP-PEG in water, representing the process of supramolecular homopolymers dynamically exchanging to form supramolecular alternating copolymers. Emission was then transformed as quenching ratio (1 − *I*/*I*_0_), where *I*_0_ was determined using a PYR-CP-PEG aqueous solution without NDI-CP-PEG (Fig. S20†). The kinetics of the formation of supramolecular alternating copolymers were determined by modeling the evolution of quenching ratio upon time by a tri-exponential function, resulting three rate constants. This suggests that there might be two different mixing processes occurring, an initial fast rate of mixing (*K*_1_) followed by two slower gradual exchange between assemblies (*K*_2_, *K*_3_) until an equilibrium is reached. By analyzing the data, as shown in Table S2,† we can conclude that increasing the temperature greatly increases the rate constants, accelerating the formation of supramolecular alternating copolymers.

### Introducing pH-responsiveness

**Fig. 6:**
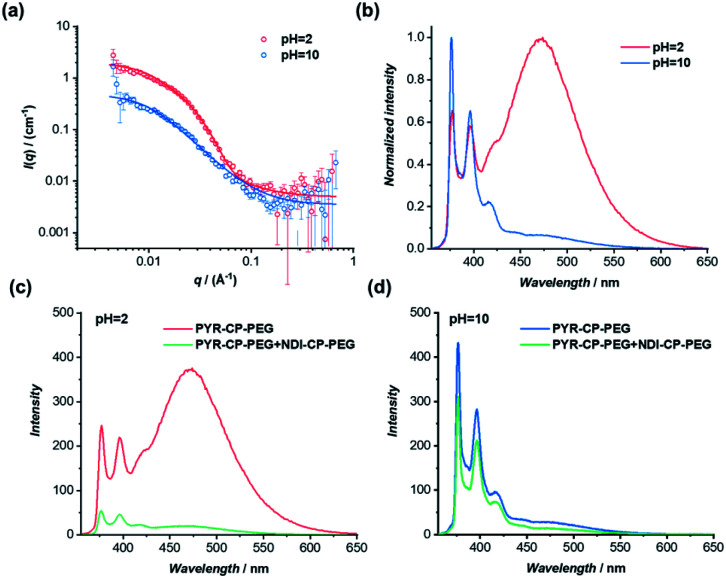
(a) Reduced SANS scattering data of PYR-CP(E_2_)-PEG at pH = 2 and pH = 10. The lines correspond to a fit to a cylinder micelle model and a Gaussian coil model, respectively; (b) normalized fluorescence spectra of PYR-CP(E_2_)-PEG at pH = 2 and pH = 10; (c) fluorescence spectra of PYR-CP(E_2_)-PEG and PYR-CP(E_2_)-PEG + NDI-CP-PEG at pH = 2; (d) fluorescence spectra of PYR-CP(E_2_)-PEG and PYR-CP(E_2_)-PEG + NDI-CP-PEG at pH = 10 (10 μM, *λ*_ex_ = 335 nm).

The dynamic nature of supramolecular polymers makes it facile to introduce stimuli-responsiveness to rationally control the sequence of the supramolecular copolymers using external stimuli. As a proof of concept, we demonstrate here the capability of reversibly controlling the transition between supramolecular alternating copolymers and supramolecular homopolymers in response to pH. Specifically, we designed and synthesized a pH-responsive conjugate by simply replacing the two tryptophans on the cyclic peptide used for PYR-CP-PEG with two glutamic acids, as PYR-CP(E_2_)-PEG (Fig. S5–S7†). The pH-responsiveness of PYR-CP(E_2_)-PEG was confirmed by SANS. As illustrated in Fig. 6a, the SANS data was fitted using a cylindrical micelle model when the pH is 2 (Table S3†); while at pH = 10, the SANS data was fitted using a Gaussian coil model (Table S4†). The change in SANS confirms the pH-responsiveness of PYR-CP(E_2_)-PEG, which forms supramolecular polymers at pH = 2 and exists as unimers at pH = 10. The pH-responsiveness could also be revealed by UV-Vis spectroscopy and fluorescence spectroscopy. As shown in Fig. S21,† a slight bathochromic shift was observed while changing the pH from 10 to 2, implying the transition of PYR from unimeric to aggregated state. A more obvious change was reflected by fluorescence spectroscopy (Fig. 6b). At pH = 10, only emission of monomeric PYR was observed, corresponding to the unimeric state, while at pH = 2, the appearance of emission ascribed to PYR excimer demonstrated the formation of supramolecular polymers.

The transition between supramolecular alternating copolymers and supramolecular homopolymers controlled by pH could be realized by co-assembling PYR-CP(E_2_)-PEG and NDI-CP-PEG. As detected by fluorescence spectroscopy in Fig. 6c, when PYR-CP(E_2_)-PEG and NDI-CP-PEG were mixed at pH = 2, a significant decrease of fluorescence (91.7%) was observed, implying the formation of supramolecular alternating copolymers. However, when the two conjugates were mixed at pH = 10, there was only a 31.5% fluorescence decrease, as PYR-CP(E_2_)-PEG is incapable of co-assembling with NDI-CP-PEG, leaving NDI-CP-PEG assembled as supramolecular homopolymers (Fig. 6d). Moreover, the transition could be reversibly tuned by simply changing the pH for several cycles (Fig. S22†).

The self-assembled supramolecular polymer sequences could be further confirmed by the binding constants between PYR-CP(E_2_)-PEG and NDI-CP-PEG at different pHs. By UV-Vis titration, the binding constant at pH = 2 was measured to be (1.05 ± 0.01) ×10^6^ M^−1^ (Fig. S23†), while at pH = 10, the binding constant decreased to (3.68 ± 0.34) × 10^4^ M^−1^ (Fig. S24†). Such a dramatic change in binding constant strongly supports the proposed transition between supramolecular alternating copolymers and supramolecular homopolymers. When the environment pH is below the p*K*_a_ of the carboxylic acid groups on the cyclic peptide, PYR-CP(E_2_)-PEG will self-assemble into supramolecular polymers. However, when the pH is higher than the p*K*_a_, the electrostatic repulsion caused by the negatively charged carboxylic acid groups will disassemble the supramolecular polymers. In this regard, PYR-CP(E_2_)-PEG and NDI-CP-PEG will co-assemble into supramolecular alternating copolymers when the pH is below p*K*_a_, When the pH is adjusted above the p*K*_a_, the supramolecular alternating copolymers will disassemble, resulting in the formation of NDI-CP-PEG supramolecular homopolymers and PYR-CP(E_2_)-PEG unimers.

## Conclusions

To conclude, we have designed and fabricated a new type of tubular supramolecular alternating copolymers based on cyclic peptide–polymer conjugates in aqueous media utilizing a combination of charge transfer interactions and multiple hydrogen bonding interactions. Different characterization techniques, including SANS, TEM, AFM, UV-Vis spectroscopy, and fluorescence spectroscopy, were used to confirm the formation of the proposed supramolecular alternating copolymers. Moreover, the noncovalent nature of supramolecular polymers allows us to monitor and even manipulate the transition between supramolecular homopolymers and alternating copolymers. Considering the fact that materials forming charge transfer complexes can show improved conductivity, it is anticipated that the tubular supramolecular alternating copolymers will act as conductive nanowires with unique core–shell structures, allowing us to fabricate well-defined supramolecular electroactive nanomaterials in aqueous media.^63,64^ On the other hand, following the same principle, more functional supramolecular alternating copolymers are expected to be designed and constructed *via* other complementary noncovalent interactions (electrostatic interactions, metal coordination interactions, and host–guest interactions, *etc.*).

## Author contributions

Qiao Song, and Sébastien Perrier conceived the project. Andrew Kerr performed the AFM experiments. Qiao Song, Jie Yang, and Sébastien Perrier analysed the data and created the figures. Stephen C. L. Hall conducted the SANS fitting. Qiao Song wrote the manuscript with input from all the authors.

## Conflicts of interest

There are no conflicts to declare.

## Supplementary Material

SC-012-D1SC02389F-s001
