# Supplementary material for: Tubular supramolecular alternating copolymers fabricated by cyclic peptide–polymer conjugates
Source: Chem Sci. 2021 Jun 3;12(26):9096–103. doi: 10.1039/d1sc02389f (PMC8261775; doi:10.1039/d1sc02389f)
Supplement: SC-012-D1SC02389F-s001 [file SC-012-D1SC02389F-s001.pdf]

Electronic Supplementary Material (ESI) for Chemical Science.
This journal is © The Royal Society of Chemistry 2021

Supporting Information

Tubular Supramolecular Alternating Copolymers Fabricated by Cyclic Peptide–Polymer Conjugate

*Qiao Song, Andrew Kerr, Jie Yang, Stephen C. L. Hall, Sébastien Perrier**

S1. Synthesis

1. Cyclic peptide H_2N-CP-N_3 (1)

Cyclic peptide H_2N-CP-N_3 (**1**) was synthesized according to procedures reported previously.[1-2]

2. Bu-NDI-BA (2)

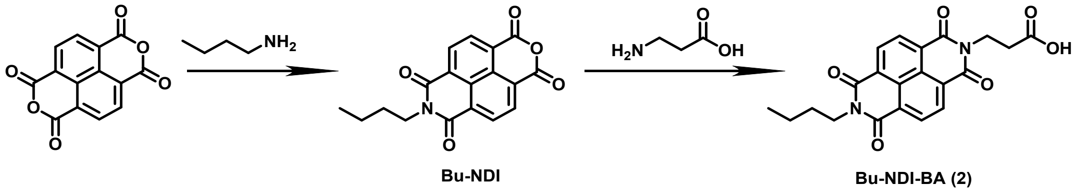

Bu-NDI-BA (**2**) was synthesized according to the literature.[3]

^{1}H-NMR (400 MHz, DMSO-*d*, ppm): 8.65 (s, 4H), 4.27 (t, 2H), 4.05 (t, 2H), 2.62 (t, 2H), 1.64 (m, 2H), 1.39 (m, 2H), 0.94 (t, 3H).

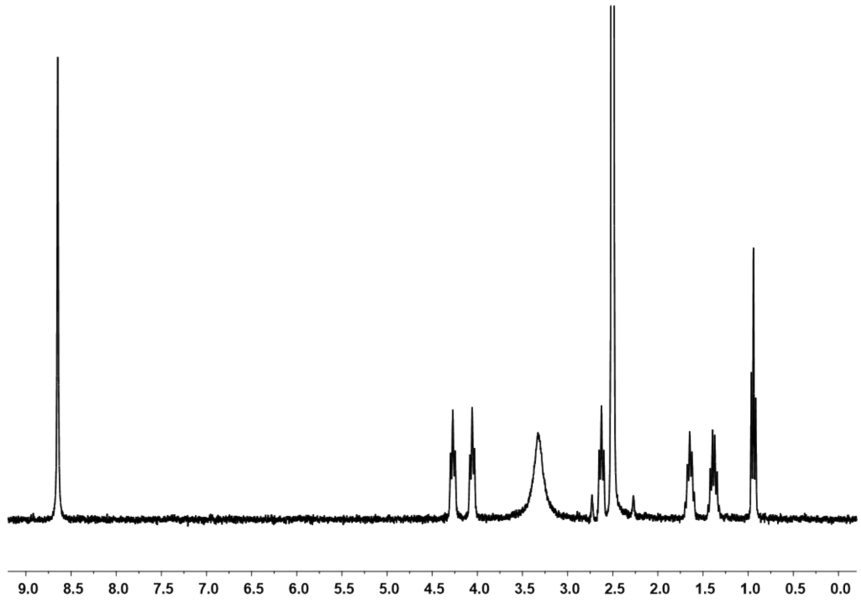

Figure S1 ^{1}H NMR spectrum of Bu-NDI-BA (**2**) (400 MHz, DMSO-*d*)

3. CH_3O-PEG-BCN (3)

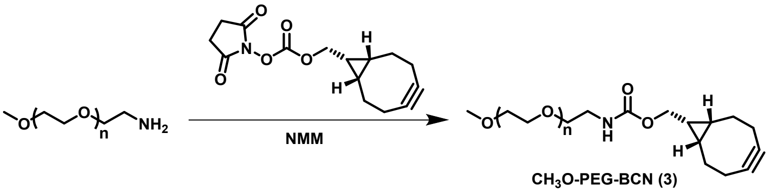

CH_3O-PEG-BCN (**3**) was synthesized according to procedures reported previously.[2]

4. PYR-CP-PEG (4)

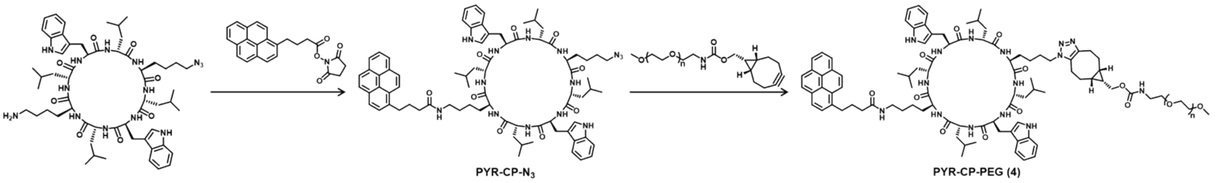

PYR-CP-PEG (**4**) was synthesized according to procedures reported previously.[2]

5. NDI-CP-PEG (5)

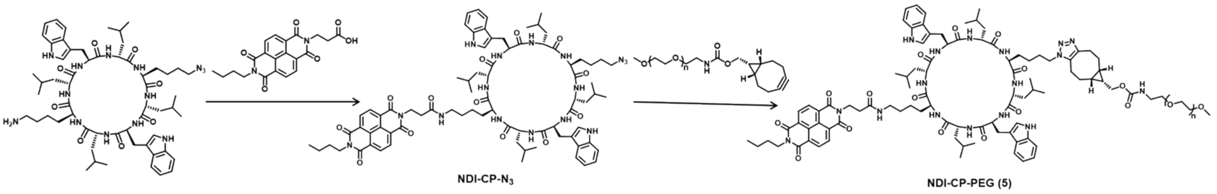

a. Synthesis of NDI-CP-N_3

H_2N-CP-N_3 **1** (20.0 mg, 0.018 mmol) and Bu-NDI-BA (21.3 mg, 0.054 mmol) were dissolved in 2 mL DMF, with the addition of HATU (20.5 mg, 0.054 mmol) and NMM (10.9 mg, 0.108 mmol). The reaction was left for 24 h. The DMF solution was then precipitated in a mixed solvent of diethyl ether: THF = 6:1 and washed twice to obtain NDI-CP-N_3 (yield: 18.0 mg). MS (ESI-ToF) (m/z): $[M+H]^+$ 1483.8 (calculated: 1483.8).

b. Synthesis of NDI-CP-PEG (5)

NDI-CP-N_3 (10 mg, 0.0067 mmol) and CH_3O-PEG-BCN **5** (53 mg, 0.0101 mmol) were dissolved in 2 mL DMF. The reaction was left for 3 days. Then the DMF solution was precipitated in cold diethyl ether. The precipitate was collected using centrifugation and dried under N_2. The resulting solid was then redissolved in 2 mL DCM and 10 mL diethyl ether was added dropwise to obtain precipitate. This process was repeated twice. Finally, NDI-CP-PEG **5** was obtained by drying under vacuum as a light brown solid (yield: 38 mg).

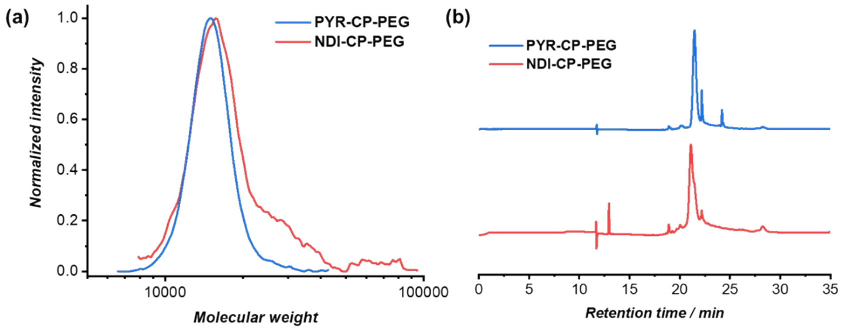

Figure S2 (a) GPC traces of PYR-CP-PEG (**4**) and NDI-CP-PEG (**5**); (b) HPLC spectra of PYR-CP-PEG (**4**) and NDI-CP-PEG (**5**) monitored by a UV detector.

6. PYR-PEG (6)

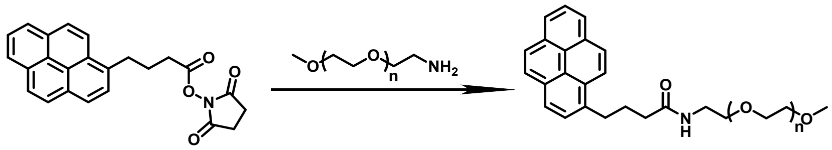

PYR-PEG (6)

PYR-PEG (**6**) was synthesized according to procedures reported previously.[2]

7. NDI-PEG (7)

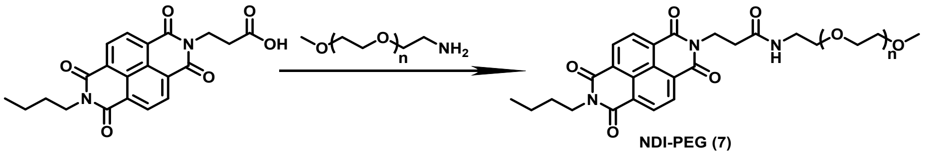

NDI-PEG (7)

CH_3O-PEG-NH_2 (50 mg, 0.01 mmol) and Bu-NDI-BA (11.8 mg, 0.03 mmol) were dissolved in 1.5 mL DMF, and HATU (11.4 mg, 0.03 mmol), NMM (6.1 mg, 0.06 mmol) was added afterwards. The reaction was left for 24 h. The DMF solution was then precipitated in a mixed solvent of diethyl ether: THF = 6:1 and washed twice to obtain NDI-PEG **7** (yield: 45 mg). ^{1}H-NMR (400 MHz, DMSO-*d*, ppm): δ = 8.68 (s, 4H), 8.04 (t, 1H), 4.27 (t, 2H), 4.04 (t, 2H), 3.68 (m, 2H), 3.51 (*br*, PEG backbone), 3.24 (s, 3H), 3.17 (m, 2H), 1.65 (m, 2H), 1.38 (m, 2H), 0.94 (t, 3H).

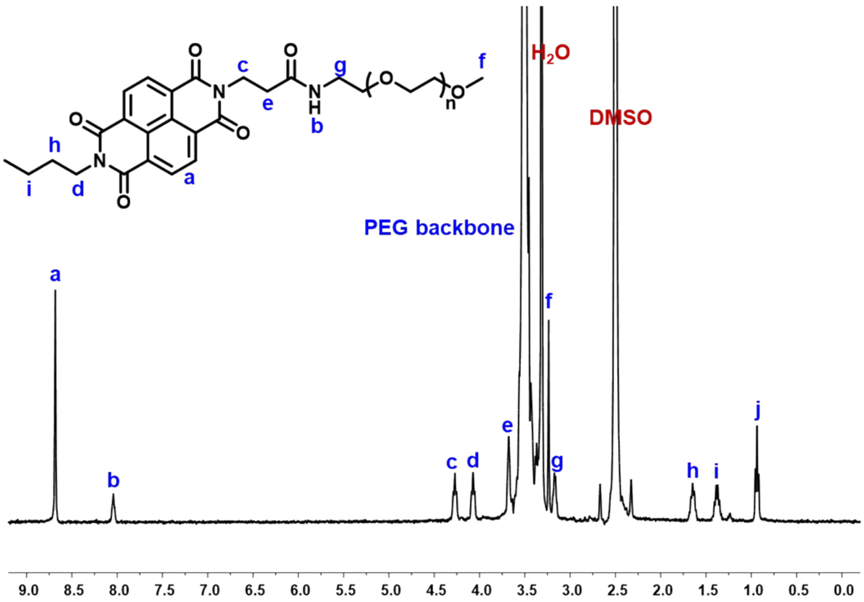

Figure S3 ^{1}H NMR spectrum of NDI-PEG (**7**) (400 MHz, DMSO-*d*)

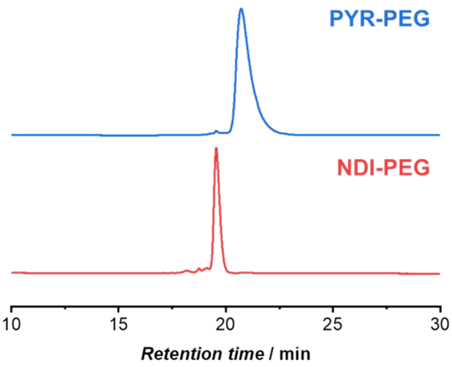

Figure S4 HPLC spectra of PYR-PEG (**6**) and NDI-PEG (**7**) monitored by a UV detector.

8. PYR-CP(E_2)-PEG (12)

a. Synthesis of cyclic peptide H_2N-CP(E_2)-N_3 (10)

The synthesis of cyclic peptide H_2N-CP(E_2)-N_3 **10** is similar to the synthesis of cyclic peptide H_2N-CP-N_3 **1**, starting from a linear peptide with the sequence of H_2N-L-Lys(N_3-pent)-D-Leu-L-Glu(OtBu)-D-Leu-L-Lys(Boc)-D-Leu-L-Glu(OtBu)-D-Leu-COOH **8**.

MS (ESI-ToF) (m/z): $[M+H]^+$ 993.6 (calculated: 993.6), $[M+Na]^+$ 1015.6 (calculated: 1015.6).

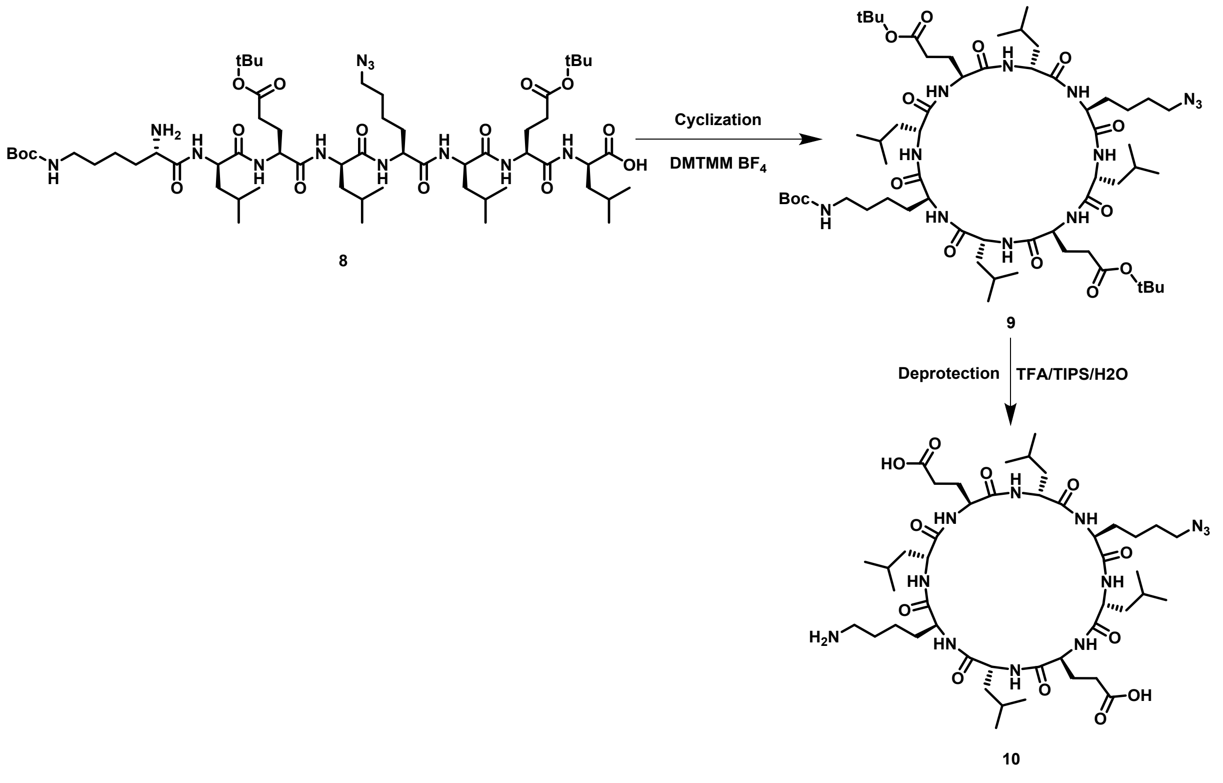

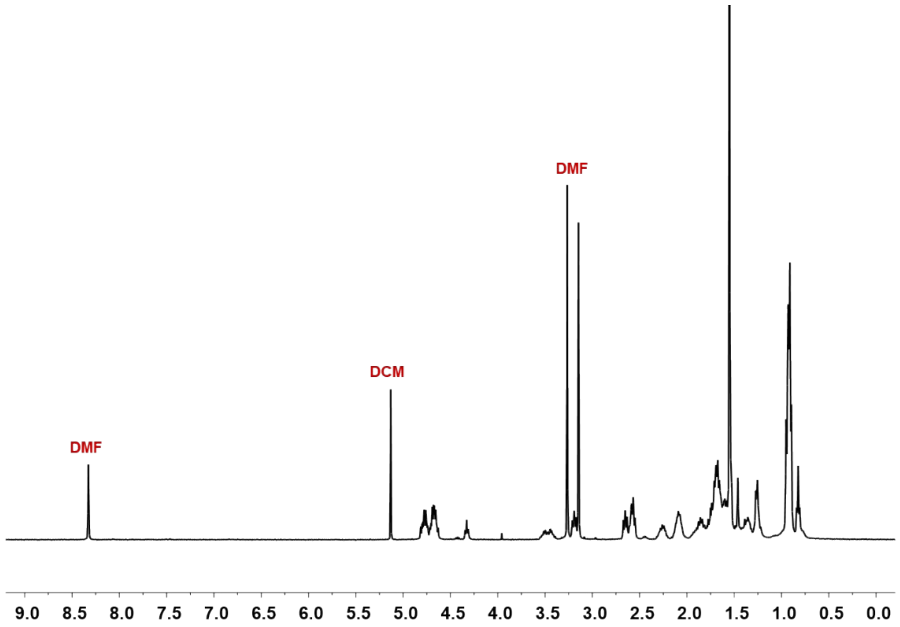

Figure S5 ^{1}H NMR spectrum of linear peptide (**8**) (400 MHz, TFA)

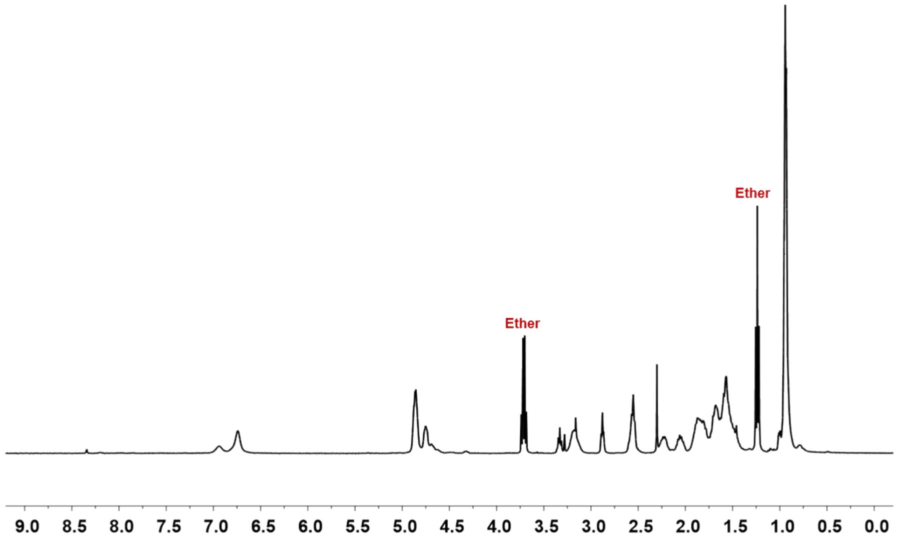

Figure S6 ^{1}H NMR spectrum of cyclic peptide H_2N-CP(E_2)-N_3 (**10**) (400 MHz, TFA)

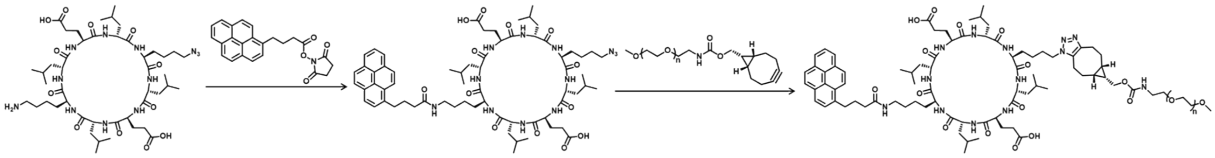

H_2N-CP(E_2)-N_3 (10) PYR-CP(E_2)-N_3 (11) PYR-CP(E_2)-PEG (12)

b. Synthesis of PYR-CP(E_2)-N_3 (11)

H_2N-CP(E_2)-N_3 **10** (10.0 mg, 0.01 mmol) and PYR-NHS (7.8 mg, 0.02 mmol) were dissolved in 1 mL DMF, with the addition of NMM (6.1 mg, 0.06 mmol). The reaction was left for 2 days. The DMF solution was then precipitated in a mixed solvent of diethyl ether: THF = 6:1 and washed twice to obtain PYR-CP(E_2)-N_3 **11** (yield: 7.0 mg).

MS (ESI-ToF) (m/z): $[M+H]^+$ 1263.7 (calculated: 1263.7).

c. Synthesis of PYR-CP(E_2)-PEG (12)

PYR-CP(E_2)-N_3 **11** (7 mg, 0.0055 mmol) and CH_3O-PEG-BCN **3** (43 mg, 0.0083 mmol) were dissolved in 1 mL DMF. The reaction was left for 3 days. Then the DMF solution was precipitated in cold diethyl ether. The precipitate was collected using centrifugation and dried under N_2. The resulting solid was then redissolved in 2 mL DCM and 10 mL diethyl ether was added dropwise to obtain precipitate. This process was repeated twice. Finally, PYR-CP(E_2)-PEG **12** was obtained by drying under vacuum as an off-white solid (yield: 21 mg).

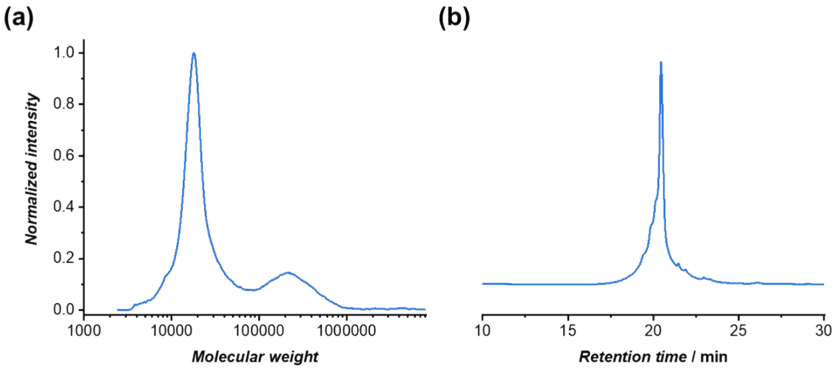

Figure S7 (a) GPC trace of PYR-CP(E_2)-PEG (**12**) (the broad peak at higher molecular weight is most likely caused by the aggregation of conjugate); (b) HPLC spectrum of PYR-CP(E_2)-PEG (**12**) monitored by a UV detector.

S3. Fabrication of tubular supramolecular polymers from PYR-CP-PEG and NDI-CP-PEG

The fabrication of tubular supramolecular polymers was realized by pre-dissolving PYR-CP-PEG or NDI-CP-PEG into a small amount of DMSO, followed by the addition of DI water, resulting clear solutions with DMSO/H_2O ratio of 5/95. In the case of supramolecular copolymers, conjugates were premixed in DMSO before adding DI water to obtain solutions with desired concentrations while keeping DMSO/H_2O ratio as 5/95. For SANS measurement, deuterated solvents (DMSO-*d* and D_2O) were used. For example, in case of solutions related to Fig. 2(c), 400 μM DMSO solutions of PYR-CP-PEG and NDI-CP-PEG were firstly prepared, respectively. The 10 μM PYR-CP-PEG and NDI-CP-PEG aqueous solutions (supramolecular homopolymer) were obtained by mixing 25 μL of the above prepared DMSO solutions and 25 μL DMSO, respectively, followed by the addition of 950 μL DI water. The 10 μM PYR-CP-PEG+NDI-CP-PEG aqueous solution (supramolecular copolymer) was prepared by mixing 25 μL PYR-CP-PEG DMSO solution and 25 μL NDI-CP-PEG DMSO solution, followed by the addition of 950 μL DI water.

SASfit software was used to fit the SANS data, using a cylindrical micelle model. SLD and brush volume values were calculated based on the molecular structures of the conjugates and solvents.

Table S1 Fitting parameters using a cylindrical micelle model by SASfit software.

Parameter	PYR-CP-PEG	NDI-CP-PEG	PYR-CP-PEG+NDI-CP-PEG
**Scale**	0.026 ± 0.016	0.062 ± 0.052	0.027 ± 0.024
**Background / cm^{-1}**	0.0008*	0.0008*	0.0015*
**Core Radius / Å**	5.1 ± 2.1	6.5 ± 1.1	6.0 ± 1.9
**Grafting Density / Å^{-2}**	0.010 ± 0.004	0.004 ± 0.001	0.007 ± 0.002
**Brush Volume / Å^3**	7413*	7413*	7413*
**SLD_{core}/ × 10^{-6} Å^{-2}**	1.61*	1.61*	1.61*
**SLD_{shell}/ × 10^{-6} Å^{-2}**	0.68*	0.68*	0.68*
**$SLD_{solvent}$/ × 10^{-6} Å^{-2}**	6.33*	6.33*	6.33*
**$R_{g,Brush}$ / Å**	35.5 ± 0.3	45.1 ± 0.3	36.4 ± 0.2
**$\chi_{v,Core\ Solvation}$**	0*	0*	0*
**Core Penetration**	0.8*	0.8*	0.8*
**Length / Å**	2000*	2000*	2000*
**χ^2/n**	4.08	14.74	6.05

* The parameters are fixed.

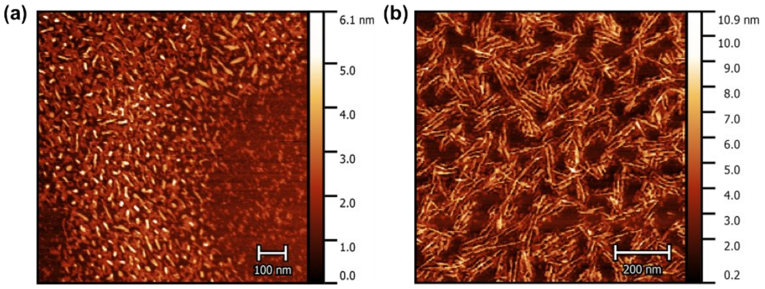

Figure S8 AFM images of PYR-CP-PEG (a) and NDI-CP-PEG (b) in water.

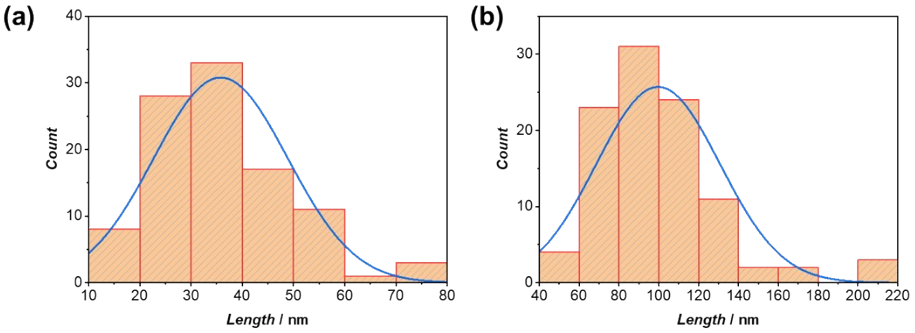

Figure S9 Length distribution of tubular supramolecular polymers assembled by PYR-CP-PEG (a) and NDI-CP-PEG(b) extracted from AFM.

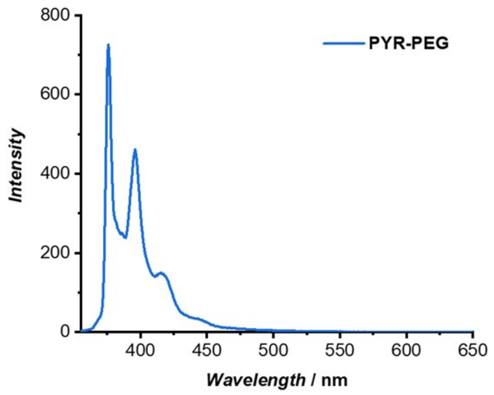

Figure S10 Fluorescence spectroscopy of PYR-PEG in water (10 μM, λ_{ex}=335 nm).

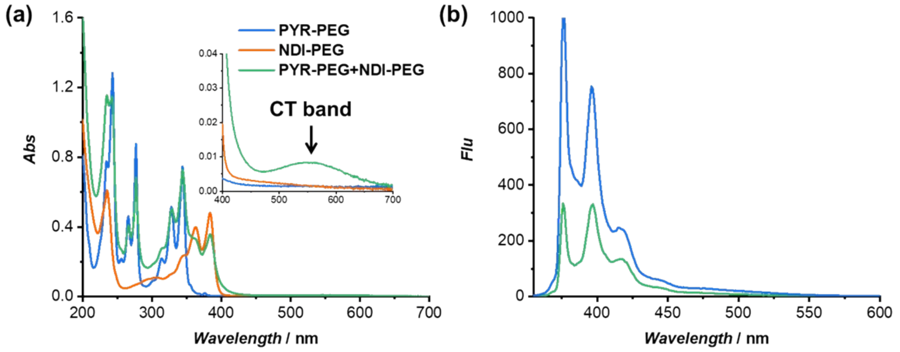

Figure S11 (a) UV-vis spectra of PYR-PEG, NDI-PEG and PYR-PEG+NDI-PEG in water; (b) Fluorescence spectra of PYR-PEG and PYR-PEG+NDI-PEG in water ([PYR-PEG]=100 μM, [NDI-PEG]=100 μM, λ_{ex}=335 nm).

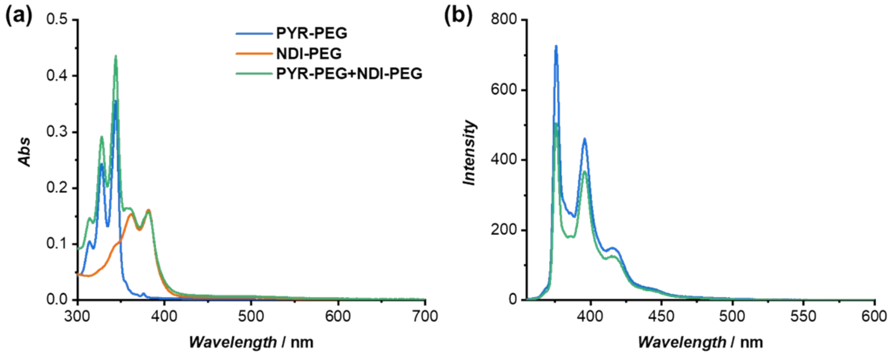

Figure S12 (a) UV-vis spectra of PYR-PEG, NDI-PEG and PYR-PEG+NDI-PEG in water; (b) Fluorescence spectra of PYR-PEG and PYR-PEG+NDI-PEG in water ([PYR-PEG]=10 μM, [NDI-PEG]=10 μM, λ_{ex}=335 nm).

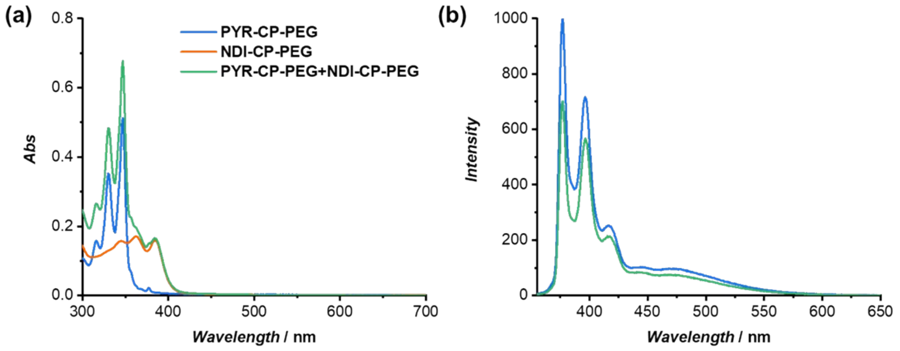

Figure S13 (a) UV-vis spectra of PYR-CP-PEG, NDI-CP-PEG and PYR-CP-PEG+NDI-CP-PEG in DMSO; (b) Fluorescence spectra of PYR-CP-PEG and PYR-CP-PEG+NDI-CP-PEG in DMSO ([PYR-CP-PEG]=10 μM, [NDI-CP-PEG]=10 μM, λ_{ex}=335 nm).

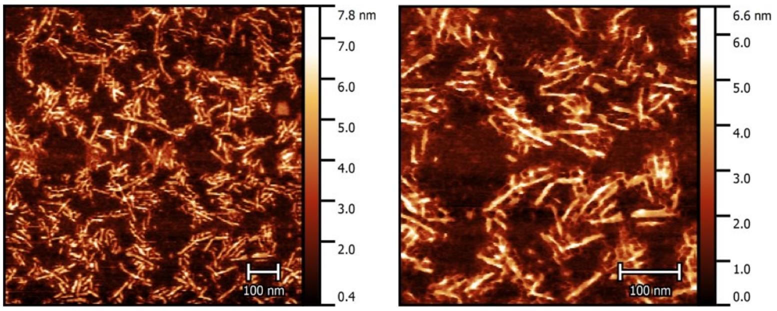

Figure S14 AFM images of the co-assembly of PYR-CP-PEG and NDI-CP-PEG.

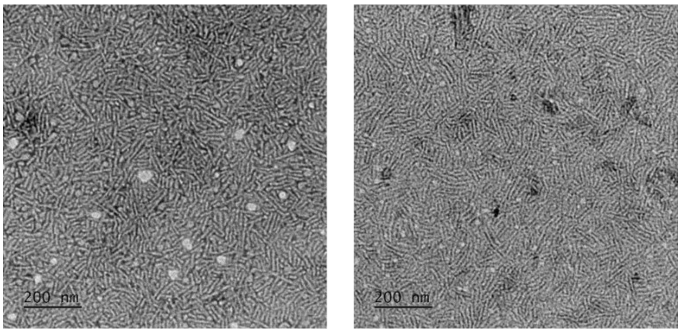

Figure S15 TEM images of the co-assembly of PYR-CP-PEG and NDI-CP-PEG.

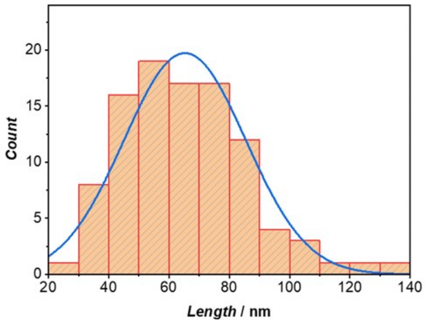

Figure S16 Length distribution of tubular supramolecular polymers co-assembled by PYR-CP-PEG and NDI-CP-PEG extracted from AFM.

S4. Binding constants measurement

a. Binding constant between PYR-PEG and NDI-PEG in water

The binding constant between PYR-PEG and NDI-PEG in water was measured by UV-vis titration. Specifically, the concentration of NDI-PEG was fixed at 40 μM, while the concentration of PYR-PEG varied from 0 to 40 μM. The absorbance at 384 nm decreased with the addition of PYR-PEG. It is assumed that the absorbance change is in linear relationship with the concentration of the CT complex in the solution. In this case, by fitting the data with a 1:1 binding model (Eq.1) using a nonlinear least-squares curve-fitting method, the binding constant between PYR-PEG and NDI-PEG could be obtained.

$$y = \frac{\alpha([D] + [A] + \frac{1}{K}) \pm \sqrt{\alpha^2([D] + [A] + \frac{1}{K})^2 - 4\alpha^2[D][A]}}{2} \qquad \text{(Eq.1)}$$

Where y is absorbance changes, $[D]$ is the concentration of donor (PYR-PEG), $[A]$ is the concentration of acceptor (NDI-PEG), K is the binding constant.

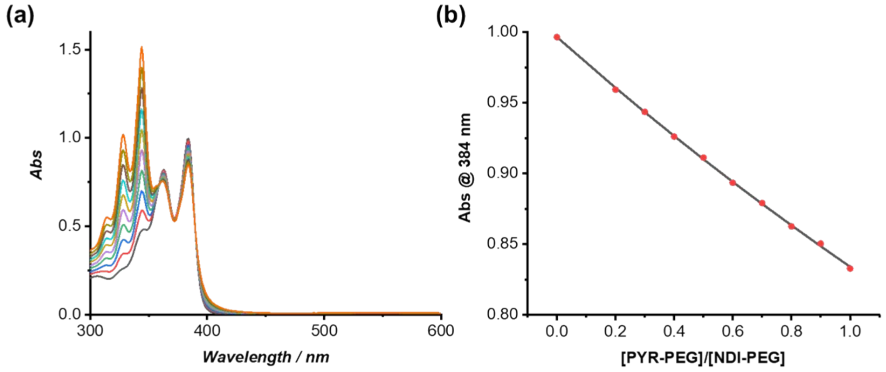

Figure S17 (a) UV-vis titration of PYR-PEG and NDI-PEG in water; (b) Evolution of absorption at 384 nm as a function of [PYR-PEG]/[NDI-PEG] and fitting using a 1:1 binding model ([NDI-PEG]=40 μM, [PYR-PEG]=0-40 μM).

b. Binding constant between PYR-CP-PEG and NDI-CP-PEG in water

The binding constant between PYR-CP-PEG and NDI-CP-PEG in water was measured by UV-vis titration. Specifically, the concentration of NDI-CP-PEG was fixed at 8 μM, while the concentration of PYR-PEG varied from 0 to 16 μM. The absorbance at 410 nm increased with the addition of PYR-PEG. It is assumed that the absorbance change is in linear relationship with the concentration of the CT complex in the solution. In this case, by fitting the data with a 1:1 binding model (Eq.1) using a nonlinear least-squares curve-fitting method, the binding constant between PYR-CP-PEG and NDI-CP-PEG could be obtained.

Note: For NDI-CP-PEG and PYR-CP-PEG, the change in absorbance at 384 nm is related to both the formation of CT complexes and the change of stacking of NDI moieties during the

copolymerization process. Instead, the absorbance at 410 nm which is contributed merely by the CT complexation was used to determine the binding constant. In case of NDI-PEG and PYR-PEG, both the absorbance at 384 nm and 410 nm can reflect the formation of CT complexes. Fitting the absorbance at 410 nm or 384 nm gives similar values.

c. Binding constant of the self-association of PYR-CP-PEG in water

The binding constant of the self-association of PYR-CP-PEG in water was measured by comparing the change of PYR unimer/excimer emission ratio at different concentrations (2-20 μM) using fluorescence spectroscopy. As the increase of PYR-CP-PEG concentration in water, the emission ratio of excimer/unimer ($\boldsymbol{I_{468}/I_{377}}$) increases. Through fitting the data with a dimerization model (Eq.2) using a nonlinear least-squares curve-fitting method, the binding constant could be obtained.

$$y = A\frac{\sqrt{8kx+1}-1}{4kx} + B\frac{4kx+1-\sqrt{8kx+1}}{4kx} \qquad \text{(Eq.2)}$$

Where x is the concentration of PYR-CP-PEG, k is the binding constant, y is $\boldsymbol{I_{468}/I_{377}}$.

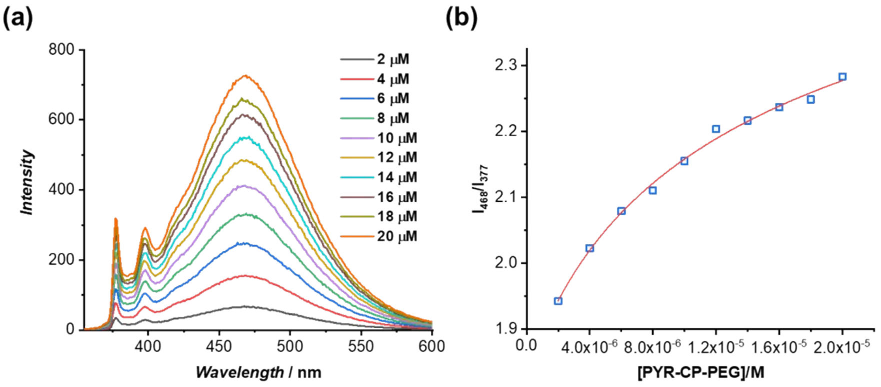

Figure S18 (a) Fluorescence spectra of PYR-CP-PEG at different concentrations in water; (b) Evolution of the ratio of the intensity at 468 nm and intensity at 377 nm as a function of PYR-CP-PEG concentration and fitting using a dimerization model.

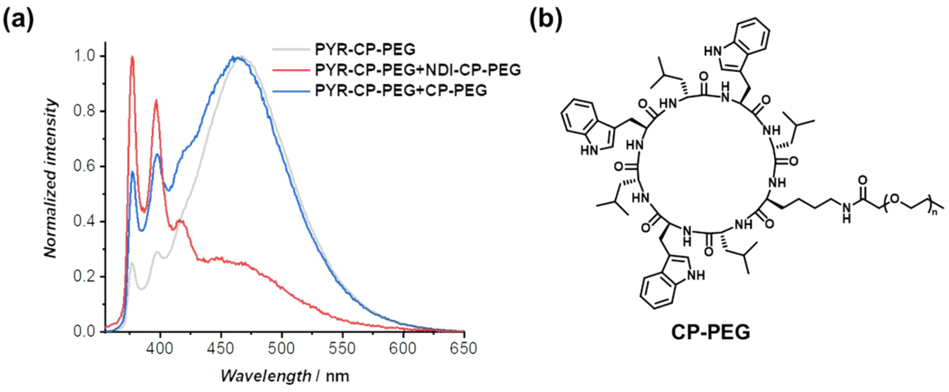

Figure S19 (a) Normalized fluorescence spectra of PYR-CP-PEG, PYR-CP-PEG+NDI-CP-PEG, and PYR-CP-PEG+CP-PEG ([PYR-CP-PEG]=10 μM, [NDI-CP-PEG]=10 μM, [CP-PEG]=10 μM, λ_{ex}=335 nm); (b) Chemical structure of CP-PEG[4].

S4. Dynamics of PYR-CP-PEG and NDI-CP-PEG in water

The mixing dynamics of PYR-CP-PEG and NDI-CP-PEG were monitored by fluorescence spectroscopy every 2 mins immediately after mixing 0.5 mL PYR-CP-PEG aqueous solution (20 μM) with 0.5 mL NDI-CP-PEG aqueous solution (20 μM) at specific temperatures (20, 50, 80 °C). The initial state was determined using PYR-CP-PEG aqueous solution (10 μM), and the fully mixed solution (final state) was prepared by premixing PYR-CP-PEG and NDI-CP-PEG in DMSO, followed by adding water to a concentration of 10 μM. The fluorescence intensity was obtained by integrating the fluorescence spectrum from 355 nm to 650 nm.

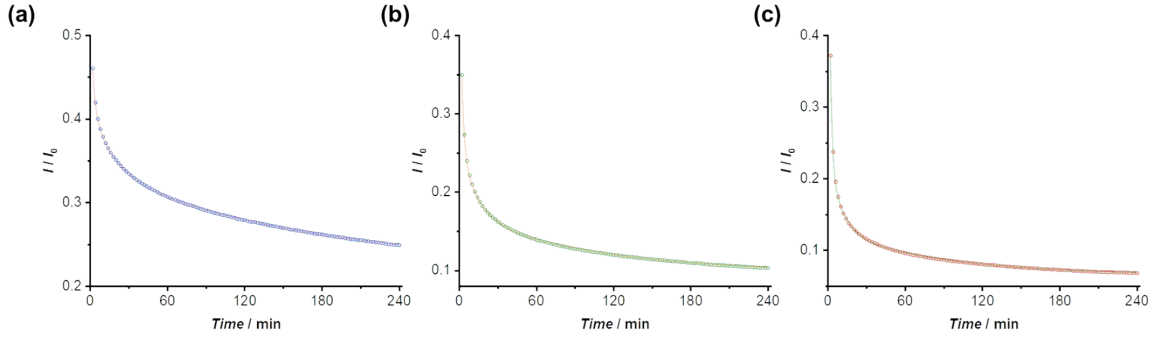

Figure S20 Evolution of quenching ratio I/I_0 over mixing time and the corresponding fitting using a tri-exponential function at 20 °C (a), 50 °C (b), and 80 °C (c).

Table S2 The rate constants of the supramolecular homopolymers dynamically exchanging to form supramolecular alternating copolymers.

Temperature/°C	K_1/h^{-1}	K_2/h^{-1}	K_3/h^{-1}	R^2
20	25.25	3.30	0.22	0.99994
50	28.79	4.34	0.55	0.99991
80	40.24	5.95	0.69	0.99987

S5. Self-assembly of pH-responsive conjugate PYR-CP(E_2)-PEG

The self-assembly of the pH-responsive conjugate PYR-CP(E_2)-PEG was conducted by pre-dissolving PYR-CP(E_2)-PEG in a small amount of DMSO, followed by the addition of either HCl aqueous solution or NaOH aqueous solution, resulting clear solutions with DMSO/H_2O ratio of 5/95 with the pH measured to be 2 and 10, respectively. For SANS measurement, deuterated solvents, and reagents (DMSO-*d*, D_2O, DCl, and NaOD) were used.

SASfit software was used to fit the SANS data, using a cylindrical micelle model. SLD and brush volume values were calculated based on the molecular structures of the conjugates and solvents.

Table S3 Fitting parameters using a cylindrical micelle model by SASfit software.

Parameter	PYR-CP(E_2)-PEG+NDI-CP-PEG (pH=2)
**Scale**	0.079 ± 0.157
**Background / cm^{-1}**	0.0008*
**Core Radius / Å**	6.0 ± 14.7
**Grafting Density / $Å^{-2}$**	0.008 ± 0.02
**Brush Volume / $Å^3$**	7413*
**SLD_{core}/ × 10^{-6} $Å^{-2}$**	1.61*
**SLD_{shell}/ × 10^{-6} $Å^{-2}$**	0.68*
**$SLD_{solvent}$/ × 10^{-6} $Å^{-2}$**	6.33*
**$R_{g,Brush}$ / Å**	27.7 ± 2.4
**$\chi_{v,Core\ Solvation}$**	0*
**Core Penetration**	0.8*
**Length / Å**	355.7 ± 0.37
**χ^2/n**	2.82

* The parameters are fixed.

Table S4 Fitting parameters using a monodisperse Gaussion coil model by SASfit software.

Parameter	PYR-CP(E_2)-PEG+NDI-CP-PEG (pH=10)
**I_0 / cm^{-1}**	0.026 ± 0.016
**R_g / Å**	0.0008*

* The parameter is fixed.

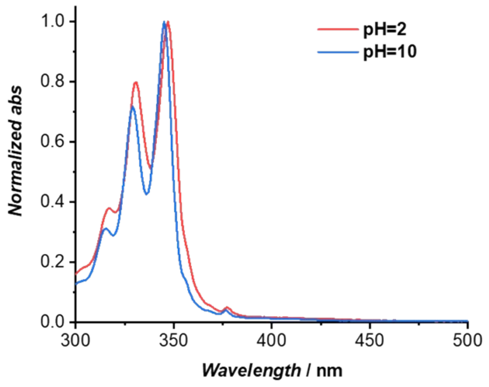

Figure S21 Normalized UV-vis spectra of PYR-CP(E_2)-PEG at pH=2 and pH=10 in water ([PYR-CP(E_2)-PEG]=10 μM).

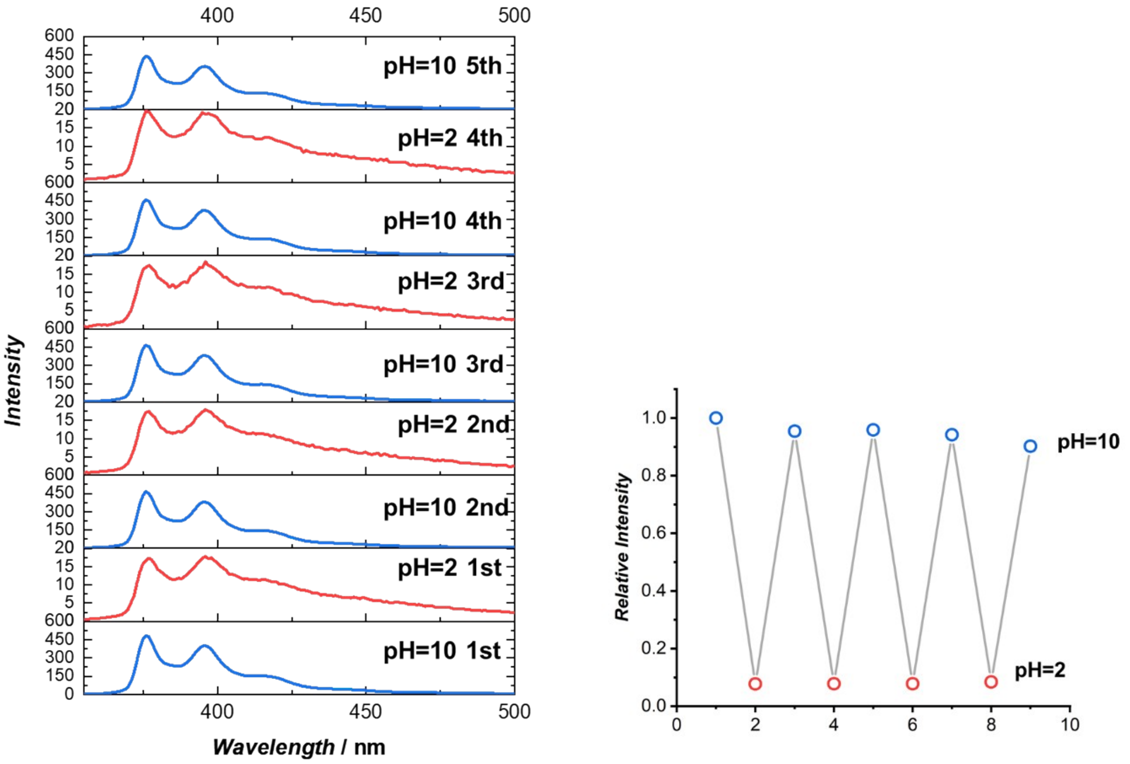

Figure S22 (a) Fluorescence spectra of PYR-CP(E_2)-PEG+NDI-CP-PEG at pH=2 and pH=10; (b) Fluorescence intensity of PYR-CP(E_2)-PEG+NDI-CP-PEG at pH=2 and pH=10.

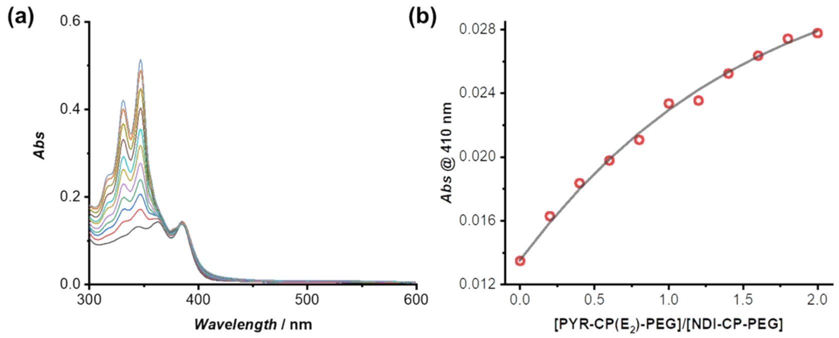

Figure S23 (a) UV-vis titration of PYR-CP(E_2)-PEG and NDI-CP-PEG in water at pH=2; (b) Evolution of absorption at 410 nm as a function of [PYR-CP(E_2)-PEG]/[NDI-CP-PEG] and fitting using a 1:1 binding model ([NDI-CP-PEG]=10 μM, [PYR-CP(E_2)-PEG]=0-20 μM).

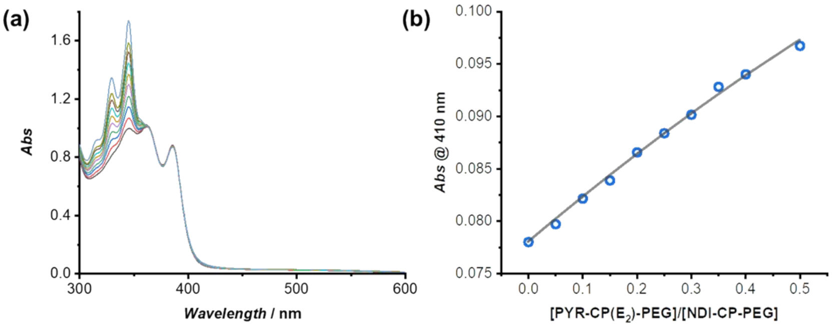

Figure S24 (a) UV-vis titration of PYR-CP(E_2)-PEG and NDI-CP-PEG in water at pH=10; (b) Evolution of absorption at 410 nm as a function of [PYR-CP(E_2)-PEG]/[NDI-CP-PEG] and fitting using a 1:1 binding model ([NDI-CP-PEG]=66.7 μM, [PYR-CP(E_2)-PEG]=0-33.3 μM).

References

1. J. Yang, J. I. Song, Q. Song, J. Y. Rho, E. D. H. Mansfield, S. C. L. Hall, M. Sambrook, F. Huang, S. Perrier, *Angew. Chem. Int. Ed.* **2020**, *59*, 8860.

2. Q. Song, S. Goia, J. Yang, S. C. L. Hall, M. Staniforth, V. G. Stavros, S. Perrier, *J. Am. Chem. Soc.* **2021**, *143*, 382.

3. M. A. Khalily, G. Bakan, B. Kucukoz, A. E. Topal, A. Karatay, H. G. Yaglioglu, A. Dana, M. O. Guler, *ACS Nano* **2017**, *11*, 6881.

4. E. D. H. Mansfield, M. Hartlieb, S. Catrouillet, J. Y. Rho, S. C. Larnaudie, S. E. Rogers, J. Sanchis, J. C. Brendel, S. Perrier, *Soft Matter* **2018**, *14*, 6320.
